# Follow-up after focal therapy in renal masses: an international multidisciplinary Delphi consensus project

**DOI:** 10.1007/s00345-016-1828-0

**Published:** 2016-04-22

**Authors:** P. J. Zondervan, P. G. K. Wagstaff, M. M. Desai, D. M. de Bruin, A. F. Fraga, B. A. Hadaschik, J. Köllermann, U. B. Liehr, S. A. Pahernik, H. P. Schlemmer, J. J. Wendler, F. Algaba, J. J. M. C. H. de la Rosette, M. P. Laguna Pes

**Affiliations:** 1 Department of Urology, AMC University Hospital, PO box 22660, 1100DD Amsterdam, The Netherlands; 2 Department of Urology, Keck School of Medicine USC, Los Angeles, CA USA; 3 Department of Biomedical Engineering and Physics, AMC University Hospital, Amsterdam, The Netherlands; 4 Department of Urology, Centro Hospitalar do Porto, Porto, Portugal; 5 Department of Urology, University Hospital Heidelberg, Heidelberg, Germany; 6 Department of Pathology, Sana Klinikum Offenbach, Offenbach, Germany; 7 Department of Urology, Otto-von-Guericke University Magdeburg, Magdeburg, Germany; 8 Department of Radiology, German Cancer Research Center, Heidelberg, Germany; 9 Department of Pathology, Fundació Puigvert, Barcelona, Spain

**Keywords:** Focal therapy, Follow-up, Renal masses, Consensus, Delphi method

## Abstract

**Purpose:**

To establish consensus on follow-up (FU) after focal therapy (FT) in renal masses. To formulate recommendations to aid in clinical practice and research.

**Methods:**

Key topics and questions for consensus were identified from a systematic literature research. A Web-based questionnaire was distributed among participants selected based on their contribution to the literature and/or known expertise. Three rounds according to the Delphi method were performed online. Final discussion was conducted during the “8th International Symposium on Focal Therapy and Imaging in Prostate and Kidney Cancer” among an international multidisciplinary expert panel.

**Results:**

Sixty-two participants completed all three rounds of the online questionnaire. The panel recommended a minimum follow-up of 5 years, preferably extended to 10 years. The first FU was recommended at 3 months, with at least two imaging studies in the first year. Imaging was recommended biannually during the second year and annually thereafter. The panel recommended FU by means of CT scan with slice thickness ≤3 mm (at least three phases with excretory phase if suspicion of collecting system involvement) or mpMRI. Annual checkup for pulmonary metastasis by CT thorax was advised. Outside study protocols, biopsy during follow-up should only be performed in case of suspicion of residual/persistent disease or radiological recurrence.

**Conclusions:**

The consensus led to clear FU recommendations after FT of renal masses supported by a multidisciplinary expert panel. In spite of the low level of evidence, these recommendations can guide clinicians and create uniformity in the follow-up practice and for clinical research purposes.

**Electronic supplementary material:**

The online version of this article (doi:10.1007/s00345-016-1828-0) contains supplementary material, which is available to authorized users.

## Introduction

Treatment of RMs has shifted from RN to nephron-sparing interventions. Conversely, increasing life expectancy has resulted in increased number of elderly patients with multiple severe comorbidities and concomitant RM. In poor surgical candidates or patients suffering from a genetic predisposition for developing multiple tumors, FT competes strongly with minimally invasive surgery [1, 2]. Interest in kidney FT has been fueled by promising reports on mid- to long-term oncological outcome combined with preservation or marginal loss of renal function [3–10].

The literature is abundant on safety and efficacy reports on CA and RFA. However, follow-up protocols are ill-defined, and the major urological associations guidelines (EAU/AUA) provide sparse guidance on the subject [2, 11, 12]. Efforts on standardization of terminology and reporting criteria by the “International Working Group on Image-Guided Tumor Ablation” have resulted in recommendations that, although non-consensually structured, are valid so far [13, 14]. However, practical guidance in terms of follow-up schedules or specific tests is not provided. With the aim of filling this gap in follow-up recommendations after FT of RMs and to provide straightforward protocols, a multidisciplinary international consensus was organized on the subject of follow-up after FT in RMs.

## Materials and methods

A consensus project based on the four Delphi method stages [15] was organized prior to and during the “8th International Symposium on Focal Therapy and Imaging in Prostate and Kidney Cancer” on June 21, 2015, in Noordwijk, The Netherlands. The four stages included:

1. Systematic literature search
  In order to assess the relevant literature, a systematic search of the PubMed database was conducted (date: January 2005–February 2015). The search focused on “renal masses” (and synonyms), “focal therapy” (and synonyms), and “follow-up” (and synonyms). The full search query and inclusion criteria to identify manuscripts are listed below (Table 1).

**Table 1:** Complete search query, filters used for the systematic literature search and inclusion criteria of the articles previous to identification of the key topics

*Search query*
(“Kidney neoplasms”[Mesh] OR kidney neoplasm*[tiab] OR kidney cancer*[tiab] OR kidney tumo*[tiab] OR kidney neoplasm*[tiab] OR kidney malignan*[tiab] OR kidney carcinoma*[tiab] OR kidney adenoma*[tiab] OR nephroma*[tiab] OR renal mass*[tiab] OR renal tumor*[tiab] OR renal tumor*[tiab] OR renal neoplasm*[tiab] OR renal cancer*[tiab] OR renal malignan*[tiab] OR renal carcinoma*[tiab] OR renal adenoma*[tiab]) AND (“Ablation Techniques”[Mesh] OR “Cryosurgery”[Mesh] OR cryotherap*[tiab] OR ablat*[tiab] OR cryoablat*[tiab] OR cryosurger*[tiab] OR RFA [tiab] OR radiofrequency ablat*[tiab] OR radio frequency ablat*[tiab] OR focal therap*[tiab]) AND (“follow-up studies”[Mesh] OR “minimally invasive surgical procedures”[Mesh] OR follow-up[tiab] OR follow-up[tiab] OR follow-up [tiab] OR CT [tiab] OR “tomography, X-ray computed”[Mesh] OR “ultrasonography”[Mesh] OR “magnetic resonance imaging”[Mesh] OR “biopsy”[Mesh] OR computed tomography[tiab] OR imaging[tiab] OR ultraso*[tiab] OR MRI[tiab] OR magnetic resonance imaging[tiab] OR biops*[tiab] OR “neoplasm recurrence, local”[Mesh] OR neoplasm persist*[tiab]) NOT (“animals”[Mesh] NOT “humans”[Mesh]) NOT (“letter” [Publication Type] OR “comment”[pt] OR “editorial”[pt])
*Filters*
Published last 10 years
English
Humans
*Inclusion criteria*
≥50 Patients included
≥24 Months follow-up
2. Defining consensus topics, formulating questions and selecting experts
  From the systematic literature review, key topics were identified, questions formulated, and online questionnaire created.
  Participants were selected based on their contribution to the literature, academic involvement or recognized expertise in the field. The multidisciplinary panel of experts in FT included urologists, radiologists, pathologists, radiation oncologists, and biomedical engineers. Biomedical engineers were asked for their involvement in new ablation technologies and diagnostics in the future.
3. Online questionnaires
  Three consecutive rounds of online questionnaires (www.surveymonkey.com) were sent to the selected experts in the period from March 29, 2015, till June 16, 2015. In the first and second rounds, the participants were encouraged to provide suggestions and feedback. Results of the previous round were incorporated in successive rounds (2nd and 3rd). Questions on which consensus was not reached were reformulated, new questions incorporated following suggestions of the participants and similar questions collated.
4. Consensus meeting
  A 6-h consensus meeting to discuss the results of the questionnaire was conducted during the 8th International Symposium on “Focal Therapy and Imaging in Prostate and Kidney Cancer” in Noordwijk, The Netherlands (www.Focaltherapy.org) from June 21, 2015, to June 23, 2015. During this last phase of the process, the results of the Web-based questionnaires were presented and discussed.

## Results

### Systematic literature search and key topics

Overall, 300 potentially eligible articles were identified by the systematic literature search. After review of titles and abstracts, 68 full-text articles were assessed for eligibility. Finally, 31 publications were selected after quality assessment (Addendum 1 in ESM). Most of these were case–control and cohort studies describing a single type of FT, comparing FT to PN outcomes or laparoscopic versus percutaneous approach. When assessing the follow-up topic, a lack of proper description of protocols was observed. Furthermore, overlap between the definitions of residual/persistent and recurrent disease frequently made it difficult to assess the results of these different outcomes separately.

Based on the lack of clarity from the literature search, we formulated five key topics. An additional query related to risk-adapted stratification follow-up showed up persistently during the online survey and was incorporated among the key topics (Table 2).

**Table 2:** Key topics identified in the literature search and pertinent questions for follow-up

Key topic	Questions
1. Definitions	What is the proper definition of persistent/residual disease? What is the proper definition of recurrent disease?
2. Follow-up intervals	What is the first time point for imaging during follow-up? What is the ideal follow-up interval? What is the ideal length of follow-up?
3. Imaging modality	Which one is the imaging modality of choice? Which alternative imaging modality in case of CKD? What are the proper CT/MRI protocols to be used? Is radiation exposure an issue during the follow-up?
4. Follow-up for metastasis	Which imaging test is recommended in the follow-up for metastasis? Which is the recommended follow-up schedule for metastasis?
5. Role of biopsy in follow-up	What is the role of biopsy in case of suspicion of residual/persistence or recurrence?
6. Risk stratification-adapted follow-up	Should follow-up be adapted to risk stratification? Which risk factors should be used?

### Participants

From 130 experts invited, 76 (58 %) accepted to participate in the project. The group consisted of 57 (75 %) urologists, 11 (14.5 %) radiologists, 5 (6.5 %) pathologists, 2 (2.6 %) engineers and 1 (1.4 %) radiation oncologist. The experience of the participants with FT of renal masses was: CA for 82 %, RFA for 67 %, HIFU for 13 %, MWA for 10 % and IRE for 17 % of the participants.

First, second and third round questionnaires were completed by 72 (95 %), 67 (88 %) and 63 (83 %) of the experts, respectively. A total of 62 (82 %) participants completed all three rounds of the online questionnaire. A panel of 12 experts, consisting of 9 urologists, 1 pathologist, 1 radiologist and 1 engineer/physicist, attended the consensus meeting in Noordwijk. Addendum 2 in ESM lists participants and their affiliations.

### Consensus

In the online questionnaires, 98 % of the participants set the cutoff for consensus at ≥80 % agreement for a specific question. A total of 51 questions were considered for consensus. Online agreement and near-agreement were reached for 19 and 6 of the questions, respectively. Online agreement was not reached in 13 questions, and 13 were exploratory with multiple possible responses. Percentage of agreement and at which round it was reached are displayed in Addendum 3 in ESM.

There was online agreement on the lack of clear recommendation on follow-up after FT, on a unique protocol after CA and RFA and on the multidisciplinary character of the follow-up protocol. During the present meeting, the composition of the follow-up team was defined as including at least one urologist, one radiologist and one pathologist with experience in FT.

### KT 1. Definition of residual/persistent and recurrent disease

Residual or persistent disease was strictly defined as the *“presence of any radiological enhancement at the first radiologic follow*-*up*”. After consensus was reached on the timing of the first radiological FU (KT 2), *the* term “at 3 months” was added (Table 3).

**Table 3:** Summary of definitions and follow-up recommendations

**1. Definitions**
Residual/persistent disease: “presence of any radiological enhancement at 3 months radiological follow-up”
Radiological recurrence: “a new (after a period of non-enhancement) enhancing or growing lesion, inside or in the margin of the ablated zone”
**2. Multidisciplinary composition of follow-up team**
At least 1 urologist, 1 pathologist and 1 radiologist (experienced in post-ablation imaging)
**3. Follow-up schedules**
*Follow-up interval:*
Minimum FU period of 5 years, preferably extended to 10 years
First FU imaging at 3 months post-treatment
A minimum of two imaging studies in the first year
Biannual imaging in the second year
Annual imaging from the third year onwards
Strongly advised not to skip on the minimum recommended number of imaging studies
*Imaging modalities*
*First option* 3-phase CT scan (non-enhanced, arterial and nephrographic/cortico-medular), slice thickness ≤3 mm, IVP phase (delayed phase) advised if suspicion of urinary tract involvement or hydronephrosis
*Second option* MRI with multiparametric protocol including at least: T1, T2, DWI, DCE
*In case of CKD 4/5* non-contrast-enhanced MRI or CEUS
*Follow-up of metastasis*
Annual examination for pulmonary metastasis, using CT thorax
Besides chest and abdomen, no other routine imaging for distant metastasis
**4. Biopsy**
Only in case of suspicion of residual disease/persistence or recurrence
**5. Risk-adapted follow-up**
Stage and grade are main determinants

**Fig. 1:**
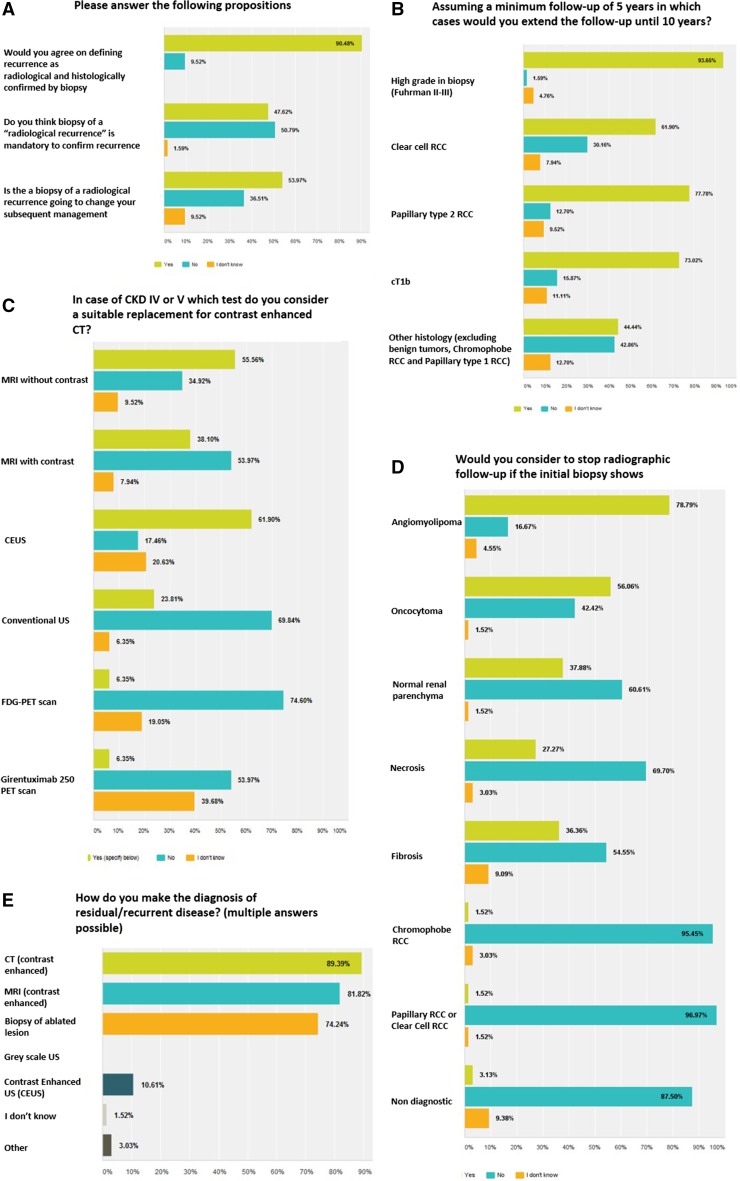
Composite figure of questions from the Delphi survey

Online questionnaires showed agreement on that “any new enhancement inside the ablated zone or in the margin of the ablated zone after a period of non-enhancement, preferably with positive biopsy” was considered as locally recurrent disease. Of the participants, 57 % considered a growing mass without enhancement as recurrence, and 1/3 of the participants would indicate a biopsy in this circumstance. Conversely, half of the participants did not consider biopsy mandatory for diagnosis of recurrence (Fig. 1a). The panel reviewed the definitions, results and suggestions and constructed a definition of radiological recurrence, as follows: “a *new (after a period of non*-*enhancement) enhancing or growing lesion, inside or in the margin of the ablated zone”* (Table 3).

Results of the online questionnaire and the panel meeting emphasized the differentiation between locally recurrent or “de novo” ipsilateral tumor (outside the treated area).

### KT 2. Follow-up intervals

The majority of the participants (74 %) recognized that contrast enhancement might persist several months after FT. During the consensus meeting, it was confirmed that a certain degree of tiny peripheral enhancement might be noted up to 6–9 months after FT that disappears subsequently. The opinion of the participants was divided at 40 % on the first timing for determination of residual disease (3 or 6 months after FT). The panel reached consensus on recommending first imaging after FT 3 months post-treatment.

No agreement was reached for number of radiological FUs in the first year. Majority (69 %) favored biannual imaging in the first year post-treatment although, when asked in consecutive round to choose between three and two imaging studies during the first year, 56 % of the participants supported imaging at 3, 6 and 12 months against 36.5 % at 6 and 12 months. Based on this apparent discordance, the panel recommended “at least” two imaging studies in the first year.

Concerning imaging FU during the second year after FT, opinions were divided between biannually and annually (55 vs 43 %, respectively). After discussion on the risk of local radiological recurrence, the panel recommended biannual imaging in the second year. From 3 years onward, annual imaging was recommended by participants’ agreement (92.5 %) and supported by the panel.

The panel stressed the lack of reliable data in the literature, the need for more frequent imaging and prolonged FU in case of aggressive pathology (high grade and cT1b) (Fig. 1b), the lack of pathological staging in FT, and that eGFR should not dictate FU intervals.

Regarding the length of the follow-up after FT, the majority of participants favored 10 years (66 %). The panel recommended a minimum follow-up term of 5 years, with the advice to prolong to 10 years because of the absence of long-term data.

### KT 3. Imaging modality

Considering participants’ response, the panel recommended three-phase CT scan (non-enhanced, arterial, and nephrographic/cortico-medular) with ≤3 mm slice thickness protocol as the imaging of choice for the follow-up after FT. Delayed or IVP phase was advised if suspicion of urinary tract involvement (leak or hydronephrosis). The second preferred imaging modality was mpMRI (T1, T2, DWI and DCE sequences, 94 % consensus) (Fig. 1d).

In case of CKD IV–V, the panel advised non-contrast-enhanced MRI as the first choice instead of non-contrast-enhanced CT. Contrast-enhanced ultrasound (CEUS) was also considered as suitable option when available and if applied by experienced hands (Fig. 1c). Of participants, 80 % considered changing imaging policy on the basis of radiation exposure. The panel emphasized that concerns on radiation exposure must not lead to skip the minimum recommended amount of imaging studies, but rather to consider a different imaging modality (e.g., MRI for young age at FT or conditions at risk of radiation accumulation).

Follow-up intervals and type of imaging did not differ between CA and RFA.

### KT 4. Follow-up for metastasis

The majority of participants (77 %) recommended regular checkup for pulmonary metastasis, at yearly intervals (89 % agreement). Based on “near-consensus” online (79 %), the panel recommended the use of CT thorax instead of X-ray thorax because of its higher sensitivity. Besides imaging of chest and abdomen, no other routine follow-up for metastasis is advised (consensus 83 %).

### KT 5. The role of biopsy during follow-up

Online agreement (85 %) was reached that post-FT biopsy should not be acquired routinely during follow-up. The participants agree (85 %) that the literature was not clear on the reliability of the biopsy to confirm residual disease. Biopsy was used by 72 % of participants to make the diagnosis or residual/recurrent disease and 2/3 recommended it for this indication. After discussion on the literature, the panel recommended biopsy during follow-up only if suspicion of residual/recurrent disease. Regarding the need to confirm radiological recurrence by biopsy, 51 % of online participants and the panel agreed that biopsies are not mandatory to confirm recurrent disease. However, the panel strongly emphasized that biopsy might be of benefit to individual patient counseling and treatment strategy.

Regarding initial biopsy results, the panel recommended to stop follow-up only in case of angiomyolipoma (Fig. 1e). In follow-up setting, opinions were divided on labeling biopsy as “non-diagnostic” in case of fibrosis, necrosis or inflammation on pathology. The panel could not find evidence in the literature to solve this question.

### KT 6. Risk stratification-adapted follow-up

There was online consensus (88 %) on “risk stratification” to guide follow-up after FT in renal masses instead of depending on procedural quality control. There was an agreement that patient and tumor factors dictate additional testing. When ranking four possible stratification factors, 1/3 of the participants found both stage and grade the most important, followed by RCC subtype. Clinical history of RCC was the least important. The panel agreed that patient and tumor factors should guide follow-up after FT, but concern was expressed on the value of adapting the currently known risk factors to the more restrictive RM population treated by FT.

## Discussion

Based on the Delphi methodology, an International multidisciplinary panel of experts discussed and formulated consensus definitions of residual/persistent and locally recurrent disease after FT of RMs. Recommendations were formulated for relevant key topics on FT follow-up including the choice of imaging modality, follow-up intervals, checkup for metastasis and the role of biopsy. Furthermore the consensus unveiled that after FT, stage and grade are the main drivers of additional testing during follow-up. The presented recommendations avoid overlap between residual and recurrent disease definitions and represent a comprehensive quality of care document.

So far, no formal recommendation process has been undertaken for follow-up of RMs after FT although the International Working Group on Image-Guided Tumor Ablation previously presented a consensus document for standardization in terminology and reporting criteria for FT in general [13, 14, 16]. EAU and AUA guidelines on RCC advise risk-adapted follow-up considering ablated tumors as intermediate- and low-risk category [2, 11, 12]. The aim of the present consensus was neither to interfere with previous documents nor to define imaging patterns of persistence or recurrence or specific recommendations on how to interpret a given test. For this purpose, a whole body of descriptive literature on post-FT imaging patterns for CT scan and MRI exists [17]. Conversely, the consensus aimed to establish clear and concise definitions of persistent and recurrent disease and to produce recommendations on which and when to apply alternative tests during FU.

Because of the absence of a well-designed comparative diagnostic study in follow-up after kidney FT and the difficulty in applying a reliable standard for comparison, we choose the Delphi method as an adequate tool to draw recommendations based on expert opinion in the medical field [15, 18]. By reformulating the questions, providing the answers of the previous rounds and narrowing the possibility for feedback, the process of achieving consensus was stimulated. Two facts strengthened the present consensus recommendations, the interdisciplinary character of the consensus Panel meeting and the high response rate reached during the three online rounds. Rather unusual in medical questionnaires, this high response rate likely reflects the strong interest and commitment of the participants [19, 20]. Furthermore, the participant’s comments forced the inclusion and discussion of a new topic: the risk-adapted follow-up. Although agreement is not necessary to reach a consensus, participants massively set a cutoff at ≥80 % as an agreement facilitating the discussion and the recommendation process.

After FT, tumor activity relies mostly on radiological evolution of the treated lesion after contrast administration. The need for repeated imaging during FT follow-up was early recognized and fully accepted. The panel agreed unanimously that a clear distinction between persistent/residual disease and local recurrence was necessary. These definitions coincide with the ones previously stated by other panels composed mainly by radiologists [13].

Follow-up schedules after FT have been erratically described and not standardized. It distillates from the literature that early contrast evaluation within the first month after treatment may not be representative of the secondary vascular necrosis and apoptotic phenomenon that ultimately condition the evolution of the ablated lesion [21, 22]. Three months was unanimously chosen as the moment for the first evaluation outside trial protocols irrespective of the ablation technology used, recognizing that the size of the lesion may be larger at this point. Rather than establishing a rigid imaging interval, the panel emitted minimal recommendations for both the period (minimum of 5 years) and interval (minimum of 2 imaging studies in the first year). Recent data suggesting that 5-year follow-up may miss up to 30 % of the recurrences in T1 tumors [23] support this recommendation especially in young or healthy patients treated by FT.

A significant percentage of patients with ablated renal masses are already known with CDK ≥ 3 at the moment of diagnosis, or will develop CKD ≥ 3 during the follow-up. The toxicity of iodine contrast agents adds to the radiation burden especially when long survival is expected [24]. Both concerns are solved by using mpMRI with or without contrast, although availability and costs may constrain the use. CEUS is the alternative test recommended by the consensus. However, this test is not available in all countries worldwide for renal tumor diagnostics or follow-up and expert interpretation is needed [25].

Data gathered by the consensus also clarifies the role of the biopsy during follow-up. Not mandatory to pronounce the diagnosis of “radiological recurrence”, the panel considered that the majority of participants used biopsy as a tool to definitive diagnosis of residual/recurrent disease and when growing mass without evidence of enhancement. In view of the scarce publications on the subject, the panel recognized that there may be a poor correlation between radiographic imaging and histopathology of the biopsy post-RFA, but probably not for Cryoablation [3, 26]. The panel recommended unanimously biopsy during FU in case of radiological suspicion of recurrence. With the limitations of expert opinion level of evidence, this seems to be the general policy in those centers practicing FT. Lastly, participants and the panel recommended stopping follow-up only in those cases with initial biopsy showing angiomyolipoma. The panel, considering the online results, recommended follow-up in case of any other biopsy results at treatment. However, in view that 56 % of respondents would stop follow-up when initial diagnostic of oncocytoma, the panel acknowledged that future studies should address this specific point. In spite of some data reporting the high reliability of oncocytoma diagnosis, the current pathology guidelines advise the use of the term “oncocytic features” specifying the preference for oncocytoma or chromophobe RCC [27].

The key topic of “risk stratification” as a guide for follow-up schedules after FT in RMs was strongly supported (88 %) in the first round of the consensus. The concept was extended in successive rounds according to participants’ feedback. It was the unanimous opinion that both patient and tumor factors should guide the risk stratification. Specifically, tumor characteristics overpowered the reliability of the procedural ablation, and tumor stage and grade were the main drivers of a “risk stratification” adapted follow-up. These two factors are universally recognized in the follow-up of any RCC, and size and stage are the most important risk factors for recurrence after kidney FT [4, 5, 28, 29]. In FT, information on grade is depending exclusively on biopsy with the consequent limitations in terms of accuracy and diagnostic yield [30]. The panel emphasized the importance of the subject and the need to strive to define “risk profiles” in the FT subpopulation of clinical renal tumors, mainly cT1a in which the classical “risk stratification” factors may require refinements.

### Limitations

In spite of its strength, the Delphi methodology is not exempt of limitations. A consensus has low level of evidence but reflects clinical practice in a topic where no RCT or well-conducted studies exist. In our specific topic, there is no literature comparing the efficacy of different follow-up schedules after FT. One should question whether such a study makes sense and will ever show a sound clinical or statistical result for an event (persistence or local recurrence) that presents scarcely in the follow-up [31].

Arguments may arise on the composition of the panel. Major difference from our expert panel when compared with previous ones on kidney FT is the high prevalence of urologists. Although there seems to be a trend from laparoscopic to percutaneous FT, it is still the responsibility and privilege of the urologist to follow up patients with localized kidney cancers after curative treatment. Thus, the panel composition and the strong recommendation on multimodal composition of the team performing the follow-up rather represent a strong than a weak point.

Lastly, the key topic “risk stratification” was explored based on participants’ feedback. While recognizing the importance of patient factors as follow-up tailoring to our surprise, previous history of RCC was ranked as the least important by almost 2/3 of the participants. Age and comorbidity were not truly explored due to complexity of the subject and the length of the survey. The panel recognized the importance of patients’ factors as determinants of a less stringent follow-up in clinical practice, but stressed the lack of a proper classification of such factors.

## Conclusions

The present consensus document defines the concepts of residual (persistent) and recurrent disease after FT of a renal mass. It recommends minimum time intervals and type of imaging to be performed during the first 5 years of follow-up. Biopsy is recommended to confirm radiological recurrence, and an extended follow-up to 10 years is advisable due to the lack of long-term data. Imaging options in case of CKD ≥3 or because of radiation exposure concerns were discussed. “risk stratification” according to patient and tumor characteristics was strongly supported by participants and the panel with so far only tumor factors (stage and grade) defined as stratifying forces.

## Electronic supplementary material

Below is the link to the electronic supplementary material.

Addendum 1 List of publications selected after quality assessment during systematic literature search (DOC 30 kb)

Addendum 2 List of consensus project participants (DOC 81 kb)

Addendum 3 List of questions posed in the 3 rounds of online surveys (DOC 155 kb)

## Abbreviations

CA: Cryoablation
CEUS: Contrast-enhanced ultrasound
CKD: Chronic kidney disease
FT: Focal therapy
HIFU: High-intensity focused ultrasound
IRE: Irreversible electroporation
KT: Key topic
MWA: Microwave
mpMRI: Multiparametric magnetic resonance imaging
PN: Partial nephrectomy
RCC: Renal cell cancer
RFA: Radio-frequency ablation
RM: Renal mass
RN: Radical nephrectomy
