# Supplementary material for: Follow-up after focal therapy in renal masses: an international multidisciplinary Delphi consensus project
Source: World J Urol. 2016 Apr 22;34(12):1657–65. doi: 10.1007/s00345-016-1828-0 (PMC5114314; doi:10.1007/s00345-016-1828-0)
Supplement: Supplementary file 1 — List of publications selected after quality assessment during systematic literature search (DOC 30 kb) [file 345_2016_1828_MOESM1_ESM.doc]

**Addendum: results systematic literature search**

(1) Aron M, Kamoi K, Remer E, Berger A, Desai M, Gill I. Laparoscopic renal cryoablation: 8-year, single surgeon outcomes. J Urol 2010; 183:889-895.

(2) Atwell TD, Callstrom MR, Farrell MA, Schmit GD, Woodrum DA, Leibovich BC, Chow GK, Patterson DE, Blute ML, Charboneau JW. Percutaneous renal cryoablation: local control at mean 26 months of followup. J Urol 2010; 184:1291-1295.

(3) Atwell TD, Schmit GD, Boorjian SA, Mandrekar J, Kurup AN, Weisbrod AJ, Chow GK, Leibovich BC, Callstrom MR, Patterson DE, Lohse CM, Thompson RH. Percutaneous ablation of renal masses measuring 3.0 cm and smaller: comparative local control and complications after radiofrequency ablation and cryoablation. AJR Am J Roentgenol 2013; 200:461-466.

(4) Balageas P, Cornelis F, Le BY, Hubrecht R, Bernhard JC, Ferriere JM, Ravaud A, Grenier N. Ten-year experience of percutaneous image-guided radiofrequency ablation of malignant renal tumours in high-risk patients. Eur Radiol 2013; 23:1925-1932.

(5) Best SL, Park SK, Yaacoub RF, Olweny EO, Tan YK, Trimmer C, Cadeddu JA. Long-term outcomes of renal tumor radio frequency ablation stratified by tumor diameter: size matters. J Urol 2012; 187:1183-1189.

(6) Desai MM, Aron M, Gill IS. Laparoscopic partial nephrectomy versus laparoscopic cryoablation for the small renal tumor. Urology 2005; 66:23-28.

(7) Gervais DA, Arellano RS, McGovern FJ, McDougal WS, Mueller PR. Radiofrequency ablation of renal cell carcinoma: part 2, Lessons learned with ablation of 100 tumors. AJR Am J Roentgenol 2005; 185:72-80.

(8) Gill IS, Remer EM, Hasan WA, Strzempkowski B, Spaliviero M, Steinberg AP, Kaouk JH, Desai MM, Novick AC. Renal cryoablation: outcome at 3 years. J Urol 2005; 173:1903-1907.

(9) Goyal J, Verma P, Sidana A, Georgiades CS, Rodriguez R. Single-center comparative oncologic outcomes of surgical and percutaneous cryoablation for treatment of renal tumors. J Endourol 2012; 26:1413-1419.

(10) Guillotreau J, Haber GP, Autorino R, Miocinovic R, Hillyer S, Hernandez A, Laydner H, Yakoubi R, Isac W, Long JA, Stein RJ, Kaouk JH. Robotic partial nephrectomy versus laparoscopic cryoablation for the small renal mass. Eur Urol 2012; 61:899-904.

(11) Hegarty NJ, Gill IS, Desai MM, Remer EM, O'Malley CM, Kaouk JH. Probe-ablative nephron-sparing surgery: cryoablation versus radiofrequency ablation. Urology 2006; 68:7-13.

(12) Ji C, Li X, Zhang S, Gan W, Zhang G, Zeng L, Yan X, Liu T, Lian H, Guo H. Laparoscopic radiofrequency ablation of renal tumors: 32-month mean follow-up results of 106 patients. Urology 2011; 77:798-802.

(13) Johnson S, Pham KN, See W, Begun FP, Langenstroer P. Laparoscopic cryoablation for clinical stage T1 renal masses: long-term oncologic outcomes at the Medical College of Wisconsin. Urology 2014; 84:613-618.

(14) Karam JA, Ahrar K, Vikram R, Romero CA, Jonasch E, Tannir NM, Rao P, Wood CG, Matin SF. Radiofrequency ablation of renal tumours with clinical, radiographical and pathological results. BJU Int 2013; 111:997-1005.

(15) Kim SD, Yoon SG, Sung GT. Radiofrequency ablation of renal tumors: four-year follow-up results in 47 patients. Korean J Radiol 2012; 13:625-633.

(16) Lawatsch EJ, Langenstroer P, Byrd GF, See WA, Quiroz FA, Begun FP. Intermediate results of laparoscopic cryoablation in 59 patients at the Medical College of Wisconsin. Journal of Urology 2006; 175:1225-1229.

(17) Leveillee RJ, Castle SM, Gorbatiy V, Salas N, Narayanan G, Morillo-Burgos G, Jorda M, Faraday MM. Oncologic outcomes using real-time peripheral thermometry-guided radiofrequency ablation of small renal masses. J Endourol 2013; 27:480-489.

(18) Matin SF, Ahrar K, Cadeddu JA, Gervais DA, McGovern FJ, Zagoria RJ, Uzzo RG, Haaga J, Resnick MI, Kaouk J, Gill IS. Residual and recurrent disease following renal energy ablative therapy: a multi-institutional study. J Urol 2006; 176:1973-1977.

(19) Ma Y, Bedir S, Cadeddu JA, Gahan JC. Long-term outcomes in healthy adults after radiofrequency ablation of T1a renal tumours. BJU Int 2014; 113:51-55.

(20) Park S, Anderson JK, Matsumoto ED, Lotan Y, Josephs S, Cadeddu JA. Radiofrequency ablation of renal tumors: intermediate-term results. J Endourol 2006; 20:569-573.

(21) Psutka SP, Feldman AS, McDougal WS, McGovern FJ, Mueller P, Gervais DA. Long-term oncologic outcomes after radiofrequency ablation for T1 renal cell carcinoma. Eur Urol 2013; 63:486-492.

(22) Ramirez D, Ma YB, Bedir S, Antonelli JA, Cadeddu JA, Gahan JC. Laparoscopic radiofrequency ablation of small renal tumors: long-term oncologic outcomes. J Endourol 2014; 28:330-334.

(23) Stern JM, Gupta A, Raman JD, Cost N, Lucas S, Lotan Y, Raj GV, Cadeddu JA. Radiofrequency ablation of small renal cortical tumours in healthy adults: renal function preservation and intermediate oncological outcome. BJU Int 2009; 104:786-789.

(24) Strom KH, Derweesh I, Stroup SP, Malcolm JB, L'Esperance J, Wake RW, Gold R, Fabrizio M, Palazzi-Churas K, Gu X, Wong C. Second prize: Recurrence rates after percutaneous and laparoscopic renal cryoablation of small renal masses: does the approach make a difference? J Endourol 2011; 25:371-375.

(25) Tanagho YS, Bhayani SB, Kim EH, Figenshau RS. Renal cryoablation versus robot-assisted partial nephrectomy: Washington University long-term experience. J Endourol 2013; 27:1477-1486.

(26) Tracy CR, Raman JD, Donnally C, Trimmer CK, Cadeddu JA. Durable oncologic outcomes after radiofrequency ablation: experience from treating 243 small renal masses over 7.5 years. Cancer 2010; 116:3135-3142.

(27) Varkarakis IM, Allaf ME, Inagaki T, Bhayani SB, Chan DY, Su LM, Jarrett TW, Kavoussi LR, Solomon SB. Percutaneous radio frequency ablation of renal masses: results at a 2-year mean followup. J Urol 2005; 174:456-460.

(28) Wah TM, Irving HC, Gregory W, Cartledge J, Joyce AD, Selby PJ. Radiofrequency ablation (RFA) of renal cell carcinoma (RCC): experience in 200 tumours. BJU Int 2014; 113:416-428.

(29) Weld KJ, Figenshau RS, Venkatesh R, Bhayani SB, Ames CD, Clayman RV, Landman J. Laparoscopic cryoablation for small renal masses: three-year follow-up. Urology 2007; 69:448-451.

(30) Schmit GD, Thompson RH, Boorjian SA, McDonald RJ, Kurup AN, Weisbrod AJ, Kor DJ, Callstrom MR, Atwell TD. Percutaneous renal cryoablation in obese and morbidly obese patients. Urology 2013; 82:636-641.

(31) Yang R, Lian H, Zhang G, Wang W, Gan W, Li X, Yan X, Zhang S, Zhao X, Guo H. Laparoscopic radiofrequency ablation with intraoperative contrast-enhanced ultrasonography for T1bN0M0 renal tumors: initial functional and oncologic outcomes. J Endourol 2014; 28:4-9.
