# Supplementary material for: Follow-up after focal therapy in renal masses: an international multidisciplinary Delphi consensus project
Source: World J Urol. 2016 Apr 22;34(12):1657–65. doi: 10.1007/s00345-016-1828-0 (PMC5114314; doi:10.1007/s00345-016-1828-0)
Supplement: Supplementary file 2 — List of consensus project participants (DOC 81 kb) [file 345_2016_1828_MOESM2_ESM.doc]

| Name | **Profession** | **Affiliated Hospital** |
| --- | --- | --- |
| Ferran Algaba | Pathology | Fundació Puigvert, Barcelona, Spain |
| Mahul Amin | Pathology | Cedars-Sinai Medical Center, Los Angeles, USA |
| Thomas Atwell | Radiology | Mayo Clinic, Rochester, USA |
| Ricardo Autorino | Urology | University Hospitals, Cleveland, USA |
| Eric Barret | Urology | Institut Montsouris, Paris, France |
| Axel Bex | Urology | NKI-AvL, Amsterdam, The Netherlands |
| Damien Bolton | Urology | University of Melbourne, Melbourne, Australia |
| Alberto Breda | Urology | Fundación Puigvert, Barcelona, Spain |
| Martijn de Bruin | Engineering/Science | AMC University Hospital, Amsterdam, The Netherlands |
| Jeffrey Cadeddu | Urology | UT Southwestern Medical Center, Dallas, USA |
| Xavier Cathelineau | Urology | Institut Montsouris, Paris, France |
| Jonathan Coleman | Urology | Memorial Sloan Kettering Cancer Center, New York, USA |
| Sebastien Crouzet | Urology | Edouard Herriot Hospital, Lyon, France |
| Andre Luis de Castro Abreu | Urology | Keck School of Medicine USC, Los Angeles, USA |
| Otto van Delden | Radiology | AMC University Hospital, Amsterdam, The Netherlands |
| Mihir Desai | Urology | Keck School of Medicine USC, Los Angeles, USA |
| Jose Dominguez-Escrig | Urology | Fundación Instituto Valenciano de Oncología, Valencia, Spain |
| Vinay Duddalwar | Radiology | Keck School of Medicine USC, Los Angeles, USA |
| Scott Eggener | Urology | University of Chicago Medical Center, Chicago, USA |
| Wouter Everaerts | Urology | UZ Leuven, Leuven, Belgium |
| Vincent Flamand | Urology | CHRU Lille, Lille, France |
| Avelino Fraga | Urology | Centro Hospitalar do Porto, Porto, Portugal |
| Ferdinand Frauscher | Radiology | University Hospital Innsbruck, Innsbruck, Austria |
| Jurgen Futterer | Radiology | Radboud MC, Nijmegen, The Netherlands |
| Michelle Gallucci | Urology | Regina Elena National Cancer Institute, Rome, Italy |
| Sangeet Ghai | Radiology | University Health Network, University of Toronto, Toronto, Canada |
| Paolo Gontero | Urology | Molinette Hospital, University of Turin, Turin, Italy |
| Rajan Gupta | Radiology | Duke University Medical Center, Durham, USA |
| Boris Hadaschik | Urology | University Hospital Heidelberg, Heidelberg, Germany |
| Markus Hohenfellner | Urology | University Hospital Heidelberg, Heidelberg, Germany |
| Christoph Klinger | Urology | Wilhelminenspital, Vienna, Austria |
| Jens Köllermann | Pathology/Urology | Sana Klinikum Offenbach, Offenbach am Main, Germany |
| Györge Kovács | Radiotherapy | University of Lübeck Medical Center, Lübeck, Germany |
| Brunolf Lagerveld | Urology | Onze Lieve Vrouwe Gasthuis, Amsterdam, The Netherlands |
| Pilar Laguna Pes | Urology | AMC University Hospital, Amsterdam, The Netherlands |
| Massimo Lazzeri | Urology | Humanitas Research Hospital, Milan, Italy |
| Benjamin Lee | Urology | Tulane University Medical Center, New Orleans, USA |
| Constantino Leonardo | Urology | University of Rome La Sapienza, Rome, Italy |
| Uwe-Bernd Liehr | Urology | Otto-von-Guericke University Magdeburg, Magdeburg, Germany |
| Uri Lindner | Urology | Kaplan Medical Center, Israel, Israel |
| Giovanni Lughezzani | Urology | Humanitas Research Hospital, Milan, Italy |
| Daniel Margolis | Radiology | UCLA Medical Center, Los Angeles, USA |
| Rodolfo Montironi | Pathology | Università Politecnica Delle Marche, Ancona, Italy |
| Aytekin Oto | Radiology | University of Chicago Medical Center, Chicago, USA |
| Sacha Pahernik | Urology | University Hospital Heidelberg, Heidelberg, Germany |
| Allan Pantuk | Urology | UCLA Jonsson Comprehensive Cancer Center, Los Angeles, USA |
| Thomas Polascik | Urology | Duke University Medical Center, Durham, USA |
| Sarah Psutka | Urology | Mayo Clinic/J.H. Stroger Jr Hospital Cook County, Chicago, USA |
| Jens Rassweiler | Urology | SLK Kliniken Heilbronn, Heilbronn, Germany |
| Ardeshir Rastinehad | Urology/Radiology | Icahn School of Medicine at Mount Sinai, New York, USA |
| Theo de Reijke | Urology | AMC University Hospital, Amsterdam, The Netherlands |
| Jean de la Rosette | Urology | AMC University Hospital, Amsterdam, The Netherlands |
| Olivier Rouvière | Radiology | Hôpital Edouard Herriot, Lyon, France |
| Peter Royce | Urology | Alfred Hospital Monash University Melbourne, Melbourne, Australia |
| Rafael Sanchez Salas | Urology | Institut Montsouris, Paris, France |
| Dilara Savci-Heijink | Pathology | AMC University Hospital, Amsterdam, The Netherlands |
| Heinz-Peter Schlemmer | Radiology | University Hospital Heidelberg, Heidelberg, Germany |
| Martin Schostak | Urology | University Hospital Magdeburg, Magdeburg, Germany |
| Christian Schwentner | Urology | UKT Tübingen University, Tübingen, Germany |
| Michiel Sedelaar | Urology | Radboud MC, Nijmegen, The Netherlands |
| Miguel Silva-Ramos | Urology | Centro Hospitalar do Porto, Porto, Portugal |
| Marc Smaldone | Urology | Fox Chase Cancer Center, Philadelphia, USA |
| Chandru Sundaram | Urology | Indiana University Hospital, Indianapolis, USA |
| Dogu Teber | Urology | University Hospital Heidelberg, Heidelberg, Germany |
| Houston Thompson | Urology | Mayo Clinic, Rochester, USA |
| Maxine Tran | Urology | Addenbrookes Hospital, Cambrigde, United Kingdom |
| Matvey Tsivian | Urology | Duke University Medical Center, Durham, USA |
| Osamu Ukimura | Urology | Keck School of Medicine USC, Los Angeles, USA |
| Massimo Valerio | Urology | CHUV, Lausanne, Suisse |
| Jochen Walz | Urology | Institut Paoli-Calmettes Cancer Centre, Marseille, France |
| Johann Wendler | Urology | University Hospital Magdeburg, Magdeburg, Germany |
| Hessel Wijkstra | Engineering/Science | AMC University Hospital, Amsterdam, The Netherlands |
| Ulrich Witzsch | Urology | Krankenhaus Nordwest Frankfurt, Frankfurt, Germany |
| Stuart Wolf | Urology | University of Michigan Health System, Michigan, USA |
| Patricia Zondervan | Urology | AMC University Hospital, Amsterdam, The Netherlands |
