# Supplementary material for: Follow-up after focal therapy in renal masses: an international multidisciplinary Delphi consensus project
Source: World J Urol. 2016 Apr 22;34(12):1657–65. doi: 10.1007/s00345-016-1828-0 (PMC5114314; doi:10.1007/s00345-016-1828-0)
Supplement: Supplementary file 3 — List of questions posed in the 3 rounds of online surveys (DOC 155 kb) [file 345_2016_1828_MOESM3_ESM.doc]

| **Question** | | | **Response** |
| --- | --- | --- | --- |
| **Consensus** | | | |
| **1.- At what percentage of agreement do you consider consensus to be reached? (Round 2)**   - 60-79% - 80% - >80% - Other | | | 7.5%  62.7%  22.4%  7.4% |
| **2.- Do you think that this Delphi consensus project is of value? (Round 2)**   - Yes - No | | | 93.9%  6.1% |
| **Protocol** | | | |
| **3.- Do you recommend different protocols for the follow-up of cryoablation versus RFA versus other ablation methods? (Round 1)**   - Yes - No - I don’t know | | | 12.5%  80.6%  6.9% |
| **4.- Do you think that there are clear recommendations on follow up after ablative treatment of kidney tumors?**  **(Round 1)**   - Yes - No - I don’t know | | | 16.7%  80.6%  2.7% |
| **5.- Do you agree that the follow-up after focal therapy in renal masses should be discussed in a multidisciplinary team? (Round 2)**   - Yes - No - I don’t know | | | 83.6%  16.4%  0.0% |
| **Definitions** | | | |
| **6.- How do you define residual disease after focal therapy in renal masses? (Round 2)**   - Presence of lesion enhancement at the first follow- up control - Presence of lesion enhancement at the end of the procedure - I don't know | 94.0%  1.5%  4.5% | | |
| **7.- Would you agree that recurrent disease can be defined as any new enhancement after a period of non-enhancement preferably with positive biopsy? (Round 3)**   - Yes - No - I don’t know | 84.1%  14.3%  1.6% | | |
| **8.- Please answer the following propositions: (Round 3)**  Would you agree on defining recurrence as radiological and histologically confirmed by biopsy   - Yes - No - I don’t know   Do you think biopsy of a “radiological recurrence” is mandatory to confirm recurrence   - Yes - No - I don’t know   Is the biopsy of a radiological recurrence going to change your subsequent management   - Yes - No - I don’t know | 90.5%  9.5%  0.0%  47.6%  50.8%  1.6%  54.0%  36.5%  9.5% | | |
| **9.- Please answer the following propositions: (Round 3)**  Local recurrence is defined as recurrence inside the ablated zone, or in the margin or the ablated zone   - Yes - No - I don’t know   Tumors in the treated kidney appearing during follow-up outside the ablated zone are defined as “the novo” tumors   - Yes - No - I don’t know | 98.4%  1.6%  0.0%  87.2%  6.4%  6.4% | | |
| **10.- Please answer the following propositions: (Round 3)**  Do you consider a persistent halo enhancement as recurrence?   - Yes - No - I don’t know   Should persistent halo after 9 months be evaluated further?   - Yes - No - I don’t know   Local recurrence is defined as new enhancing node in the ablated zone irrespective of the size   - Yes - No - I don’t know   Do you consider a growing mass even without any type of enhancement as recurrence?   - Yes - No - I don’t know | 17.5%  61.9%  20.6%  71.4%  14.3%  14.3%  87.3%  7.9%  4.8%  57.1%  30.2%  12.7% | | |
| **11.- What do you do when a lesion does not enhance but does not regress in size? (Round 3)**   - Active treatment - Biopsy - Nothing but imaging in follow-up - I don't know | 0.0%  33.3%  63.5%  3.2% | | |
| **Follow-up term/interval** | | | |
| **12.- Which follow-up term do you recommend after focal treatment of renal masses? (Round 2)**   - Up to 5 years - Up to 10 years - I don’t know | Cryoablation / RF  29.9% / 31.3%  65.7% / 65.7%  4.5% / 3.0% | | |
| **13.- Do you think that there is enough evidence that persistent contrast enhancement after ablative treatment can last for several months? (Round 2)**   - Yes - No - I don’t know | 74.2%  13.7%  12.1% | | |
| **14.- Persistent halo enhancement has been described up to 6-9 months after ablation. At what time point should the presence/absence of residual disease be determined? (Round 2)**   - 3 months - 6 months - 1 year - I don’t know | Cryoablation / RF  41.8% / 41.8%  41.8% / 43.3%  11.9% / 10.4%  4.5% / 4.5% | | |
| **15.- Considering that persistent enhancement has been described in literature up to 6-9 months post focal treatment. In the case of persistent enhancement at 6-9 months after ablative treatment, and negative biopsy, what would you do? (Round 2)**   - Follow by imaging - Salvage treatment - I don't know | 86.4%  10.6%  3.0% | | |
| **16.- Not considering research purposes. At what time point do you recommend the FIRST follow-up imaging after focal therapy of renal masses? (Round 2)**   - 1 month - 3 months - 6 months - 1 year - I don’t know | Cryoablation / RF  3.0% / 4.5%  85.1% / 85.1%  9.0% / 7.5%  0.0% / 0.0%  2.9% / 2.9% | | |
| **17.- What is the minimal interval that you recommend for follow-up imaging in the FIRST YEAR after focal therapy of renal masses? (Round 2)**   - Every 3 months - Biannually - I don’t know | Cryoablation / RF  25.4% / 26.9%  70.2% / 68.7%  4.4% / 4.5% | | |
| **18.- Assuming there is no contrast enhancement in any of the follow-up imaging, what schedule will you choose during the first year? (Round 3)**   - Imaging at 3, 6 and 12 months - Imaging at 3, 6, 9 and 12 months - Imaging at 6 and 12 months - I don't know | 55.6%  7.9%  36.5%  0.0% | | |
| **19.- If there were no previous signs of radiological recurrence, at what minimal interval do you recommend for follow-up imaging in the SECOND YEAR after focal therapy of renal masses? (Round 2)**   - Biannually - Annually - I don’t know | Cryoablation / RF  55.2% / 55.2%  43.3% / 43.3%  1.5% / 1.5% | | |
| **20.-Assuming absence of radiological signs of recurrence at the end of the first year after FT, why would you recommend a MINIMAL radiological control biannually? (Round 3)**  Because aggressive pathology in initial biopsy   - Yes - No - I don’t know   Because chances of recurrence are higher during the first 2 years   - Yes - No - I don’t know   Would biannual imaging be reasonable in year 2 assuming first year imaging is negative?   - Yes - No - I don’t know | 84.1%  14.3%  1.6%  82.5%  12.7%  4.8%  76.2%  12.7%  11.1% | | |
| **21.- If there were no previous signs of radiological recurrence, at what minimal interval do you recommend follow-up imaging in the THIRD YEAR after focal therapy of renal masses? (Round 2)**   - Biannually - Annually - I don’t know | Cryoablation / RF  7.5% / 7.5%  89.6% / 89.6%  2.9% / 2.9% | | |
| **22.- If there were no previous signs of radiological recurrence, at what minimal interval do you recommend follow-up imaging from 3 YEARS ONWARDS after focal therapy of renal masses? (Round 2)**   - Biannually - Annually - I don’t know - Other | Cryoablation / RF  3.0% / 3.0%  92.5% / 92.5%  3.0% / 3.0%  1.5% / 1.5% | | |
| **23- Assuming a minimum follow-up of 5 years in which cases would you extend the follow-up until 10 years? (Round 3)**  High grade in biopsy (Fuhrman II-III)   - Yes - No - I don’t know   Clear cell RCC   - Yes - No - I don’t know   Papillary type 2 RCC   - Yes - No - I don’t know   cT1b   - Yes - No - I don’t know   Other histology (excluding benign tumors, Chromophobe RCC and Papillary type 1 RCC)   - Yes - No - I don’t know | 93.7%  1.6%  4.7%  61.9%  30.2%  7.9%  77.8%  12.7%  9.5%  73.0%  15.9%  11.1%  44.4%  42.9%  12.7% | | |
| **24.- Is eGFR a determinant for the follow-up interval? (Round 1)**   - Yes - No - Other | Cryoablation / RF  38.5% / 38.5%  52.3% / 52.3%  9.2% / 9.2% | | |
| **Imaging modalities** | | | |
| **25.- Which imaging modalities do you recommend for the follow-up of focal therapy in renal masses? Multiple answers possible. (Round 1)**   - Contrast enhanced CT - MRI - US - CEUS - PET-CT - I don’t know | Cryoablation / RF  84.9% / 87.9%  71.2% / 68.2%  13.6% / 12.1%  15.2% / 15.2%  4.6% / 4.6%  4.6% / 4.5% | | |
| **26.- Which CT protocol do you recommend in the follow up of FT in renal masses? (Round 3)**   - 4 phases (non enhanced, arterial, cortico-medular or nefrographic and excretory) - 3 phases (non enhanced, arterial and cortico-medular or nefrographic) - 2 phases (non enhanced and arterial) - 2 phases (non enhanced and cortico-medular or nefrographic) - 2 phases (arterial and cortico-medular or nefrographic) - I don’t know | 28.6%  49.2%  6.4%  4.8%  1.6%  9.4% | | |
| **27.- In clinical practice, which CT slice thickness do you recommend for the follow-up of focal therapy in renal masses? (Round 2)**   - ≤ 3 mm - 5 mm - > 5 mm - I don’t know | Cryoablation / RF  84.9% / 84.9%  10.6% / 10.6%  1.5% / 1.5%  3.0% / 2.0% | | |
| **28.- If MRI is used, which MRI sequences do you recommend for the follow-up of focal therapy in renal masses? Multiple answers possible. (Round 2)**   - Multi parametric protocol - Other - I don’t know | Cryoablation / RF  94.0% / 94.0%  3.0% / 3.0%  3.0% /3.0% | | |
| **29.- Is eGFR a determinant for the follow-up imaging modality? (Round 1)**   - Yes - No - I don’t know | Cryoablation / RF  78.8% / 80.3%  10.6% / 7.6%  10.6% / 12.1% | | |
| **30.- In case of CKD grade IV or V would you apply an imaging test other than CT or MRI? (Round 2)**   - Yes - No - I don’t know | Cryoablation / RF  37.3% / 37.3%  46.3% / 46.3%  16.4% / 16.4% | | |
| **31.- In case of CKD IV or V which test do you consider a suitable replacement for contrast enhanced CT. (Round 3)**  MRI without contrast   - Yes - No - I don’t know   MRI with contrast   - Yes - No - I don’t know   CEUS   - Yes - No - I don’t know   Conventional US   - Yes - No - I don’t know   FDG-PET scan   - Yes - No - I don’t know   Girentuximab 250 PET scan   - Yes - No - I don’t know | 55.6%  34.9%  9.5%  38.1%  54.0%  7.9%  61.9%  17.5%  20.6%  23.8%  69.8%  6.4%  6.4%  74.6%  19.0%  6.4%  54.0%  39.6% | | |
| **32.- In young people treated by FT, does radiation exposure play a role in your imaging policy? (Round 3)**   - Yes - No - I don’t know | 77.8%  20.6%  1.6% | | |
| **33.- Do you ever consider changing your imaging policy on the basis of radiation exposure? (Round 3)**   - Yes - No - I don’t know | 79.4%  19.1%  1.5% | | |
| **Biopsy** | | | |
| **34.- Do you recommend post-ablation biopsies in the follow-up of focal therapy in renal masses ( Round 2)**   - Yes, routinely - Only in case of suspicion of persistence/recurrence - No - I don’t know - Other | Cryoablation / RF  3.0% / 4.5%  85.1% / 84.9%  7.5% / 6.1%  0.0% / 0/0%  4.4% / 4.5% | | |
| **35.- Do you think that the literature is clear on the reliability of biopsy for confirming residual disease? (Round 2)**   - Yes - No - I don’t know | 13.6%  84.9%  1.5% | | |
| **36.- How do you make the diagnosis of residual/recurrent disease? Multiple answers possible. (Round 1)**   - CT (contrast enhanced) - MRI (contrast enhanced) - Biopsy of the ablated lesion - Grey scale US - CEUS - I don’t know - Other | 89.4%  81.8%  72.2%  0.0%  10.6%  1.5%  3.0% | | |
| **37.- Do you recommend the use of biopsies to make the diagnosis of residual/recurrent disease? (Round 2)**   - Yes - No - I don’t know | 67.2%  31.3%  1.5% | | |
| **38.- Should be biopsy routinely indicated for residual/new enhancement with the knowledge that there is a high incidence of false-negative rates and treatment may be based on imaging alone? (Round 3)**   - Yes - No - I don’t know | 60.3%  38.1%  1.6% | | |
| **39.- Would you consider to stop radiographic follow-up if the initial biopsy shows: (Round 2)**  Angiomyolipoma   - Yes - No - I don’t know   Oncocytoma   - Yes - No - I don’t know   Normal renal parenchyma   - Yes - No - I don’t know   Necrosis   - Yes - No - I don’t know   Fibrosis   - Yes - No - I don’t know   Chromophobe RCC   - Yes - No - I don’t know   Papillary RCC or Clear Cell RCC   - Yes - No - I don’t know   Non diagnostic   - Yes - No - I don’t know | 78.8%  16.7%  4.5%  56.1%  42.4%  1.5%  37.9%  60.6%  1.5%  27.3%  69.7%  3.0%  36.4%  54.6  9.0%  1.5%  95.5%  3.0%  1.5%  97.0%  1.5%  3.1%  87.5%  9.4% | | |
| **40.- In the setting of FT in kidney tumors, do you consider the following diagnostics as Non-diagnostic biopsy? (Round 3)**  Fibrosis   - Yes - No - I don’t know   Normal renal parenchyma   - Yes - No - I don’t know   Necrosis   - Yes - No - I don’t know   Inflamation   - Yes - No - I don’t know | 50.8%  44.4%  4.8%  73.0%  22.2%  4.8%  57.1%  36.5%  6.4%  46.0%  44.4%  9.5% | | |
| **Metastasis** | | | |
| **41.- During follow-up after focal treatment in renal masses do you recommend checking for pulmonary metastasis regularly? (Round 2)**   - Yes, routinely - Only if high risk according to integrated prognostic system - No | | 77.3%  21.2%  1.5% | |
| **42.- If you want to rule out pulmonary metastasis during follow-up after focal therapy, which imaging modality do you recommend? Multiple answers possible. (Round 2)**   - X-thorax - CT-thorax - I don’t know | | 25.7%  77.3%  3.0% | |
| **43.- If screening for pulmonary metastasis is performed, at what minimal interval should screening take place? (Round 2)**   - Biannually - Annually - I don’t know | | 12.1%  88.9%  0.0% | |
| **44.- Do you check for any metastasis routinely other than chest and abdomen? (Round 3)**   - Yes - No - I don't know | | 12.7%  82.5%  4.8% | |
| **45.- For routine checking of pulmonary metastasis during FU of FT for RCC, which of the following do you use? (Round 3)**   - A general guideline (local or global) for follow-up of treatment of RCC according to risk profiles An adapted schedule for focal treatment? - I don't know | | 87.2%  6.4%  6.4% | |
| **46.- Would you be satisfied if CT-thorax was recommended as standard modality to rule out pulmonary metastasis during follow-up after FT of renal masses? (Round 3)**   - Yes - No - I don’t know | | 79.4%  17.5%  3.1% | |
| **Risk stratification** | | | |
| **47.- Do you think an individual patient risk-stratification should be used to determine the duration of follow-up? (Round 1)**   - Yes - No - I don’t know | | 87.7%  9.2%  3.1% | |
| **48.- Is your follow-up dependent on quality control during ablation (procedural monitoring of ablation zone)? (Round 2)**   - Yes - No - I don’t know | | 41.8%  53.7%  4.5% | |
| **49.- Would you accept a minimum follow-up protocol for CRYO and RF ablation with additional testing based on: (Round 3)**  Individual patients factors (e.g age or previous history of RCC)   - Yes - No - I don’t know   The tumor size and histology of the biopsy   - Yes - No - I don’t know | | 95.2%  1.6%  3.2%  96.8%  0.0%  3.2% | |
| **50.- What do you think is the most important factor on which to base the follow-up protocol? (Round 3)**   - Tumor characteristics - Reliability of ablation (procedural monitoring, eventually CT control at the end of the procedure, intraoperative factors) - Published results on the different technologies - Other - I don't know | | 52.4%  33.3%  9.5%  0.0%  4.8% | |
| **51.- If you should propose a risk stratification for kidney tumors treated by FT what would you take into account? Please rank the following form 1 (most important) to 4 (least important). (Round 3)**  Clinical stage   - 1 - 2 - 3 - 4 - I don’t know   Grade   - 1 - 2 - 3 - 4 - I don’t know   RCC Subtype   - 1 - 2 - 3 - 4 - I don’t know   Clinical history of RCC   - 1 - 2 - 3 - 4 - I don’t know | | 36.5%  14.3%  30.2%  11.1%  7.9%  31.8%  34.9%  19.1%  6.4%  7.8%  9.5%  38.1%  34.9%  6.4%  11.1%  4.3%  4.8%  4.8%  63.5%  12.6% | |
